# Chemical Composition and Biological Activities of the Essential Oils of *Leptospermum petersonii* and *Eucalyptus gunnii*

**DOI:** 10.3389/fmicb.2020.00409

**Published:** 2020-04-15

**Authors:** Lucia Caputo, Antonella Smeriglio, Domenico Trombetta, Laura Cornara, Greg Trevena, Marco Valussi, Florinda Fratianni, Vincenzo De Feo, Filomena Nazzaro

**Affiliations:** ^1^Department of Pharmacy, University of Salerno, Fisciano, Italy; ^2^Department of Chemical, Biological, Pharmaceutical and Environmental Sciences, University of Messina, Messina, Italy; ^3^Department for the Earth, Environment and Life Sciences, School of Mathematical, Physical and Natural Sciences, University of Genoa, Genoa, Italy; ^4^Essentially Australia, Byron Bay, NSW, Australia; ^5^European Herbal and Traditional Medicine Practitioners Association, Norwich, United Kingdom; ^6^Institute of Food Sciences, CNR-ISA, Italian National Research Council, Avellino, Italy

**Keywords:** Myrtaceae, essential oil, phytochemical profile, antibacterial activity, biofilm, phytotoxic activity

## Abstract

The aim of this study was to characterize the chemical composition and to evaluate the antimicrobial and phytotoxic properties of the essential oils (EOs) obtained from leaves of *Leptospermum petersonii* chemotype “Variety B” and *Eucalyptus gunnii*, native to Australia. Geranyl acetate, γ-terpinene, geraniol, terpinolene, α-pinene, *p*-cimene, and linalool were the main components in *L. petersonii* EO, confirming also the existence of several chemotypes in such taxa; on the other hand, 1,8-cineole, *trans*-sabinene hydrate acetate, globulol, longicyclene, terpinolene, and camphene were present in major amounts in the *E. gunnii* EO. Chemical analysis of *L. petersonii* revealed that it belongs to the variety “B.” *E. gunnii* EO showed good antibacterial activity, with an MIC of 0.5 and 2 μg/mL against *Staphylococcus aureus*, and *Pectobacterium carotovorum*, respectively. The activity of *E. gunnii* EO was stronger than *L. petersonii* EO, whose maximum MIC reached 5 μg/mL. *E. gunnii* and *L. petersonii* EOs were particularly effective in inhibiting the biofilm formation by *S. aureus*, already at a concentration of 0.01 μg/mL. The other strains were resistant to both EOs up to a dose of 0.05 μg/mL. The maximum inhibition on biofilm formed by *P. carotovorum* was recorded for *E. gunnii* EO, reaching a value of 93.12% at 1.0 μg/mL. This is the first manuscript which studies the biofilm inhibition by EOs and evaluates their effects on biofilm metabolism. Both EOs were more effective against *P. carotovorum*. In addition, even though *L. petersonii* EO 0.1 μg/mL was unable to inhibit biofilm formation by *Escherichia coli*, it decreased the metabolic activity of the biofilm to 78.55% compared to control; furthermore, despite it inducing a relatively low inhibition (66.67%) on biofilm formation, it markedly affected metabolic activity, which decreased to 16.09% with respect to the control. On the contrary, *L. petersonii* EO 0.5 μg/mL induced a 79.88% inhibition of *S. aureus* biofilm, maintaining a high metabolic activity (90.89%) compared to the control. Moreover, this EO showed inhibitory activity against radical elongation of *Solanum lycopersicum* and the germination of radish. On the contrary, *E. gunnii* EO showed no phytotoxic activity.

## Introduction

*Leptospermum petersonii* F.M. Bailey and *Eucalyptus gunnii* Hook.f. belong to the Myrtaceae family. This plant family comprises of at least 3000 species widely distributed in several tropical and warm-temperate areas, such as Australia and Central and South America (Mabberley, 1997). Many EOs produced by the Myrtaceae species have been reported for their insecticidal, nematicidal, anti-inflammatory, and antifungal activities and are used as antimicrobial agents in cosmetic products (Lis-Balchin et al., 2000; Lee et al., 2004; Park et al., 2011).

*Leptospermum petersonii*, commonly known as Australian Rose (lemon-scented tea-tree), is a rare little tree, naturally occurring in lowland or floodplain areas in Northern New South Wales. Previous literature proposed the existence of three chemical varieties of this species, based on the composition of its EO (Brophy et al., 2000). At present, there is no knowledge of indigenous uses of *L. petersonii.* However, other species of the same genus are known to be used in traditional medicine, such as *Leptospermum flavescens* Sm., used in Malaysia as a way to relieve stomach disorders and menstrual disorders (Riley, 1994), and the New Zealand species *Leptospermum scoparium* J. R. Forst. and G. Forst., used by Maori as a food and as a traditional remedy to alleviate coughs (Brooker et al., 1987; Crowe, 1997). The odor of common *L. petersonii* EO is described as “extremely pleasant and lemony” (Nuadha, 2011). *L. petersonii* (FM. Bailey) EO showed insecticidal activity against the diamond back moth *Plutella xylostella* (L.), reducing the feeding and development of larval stages on broccoli leaves and oviposition in adult stage. In light of this, it could be used as an alternative insecticidal strategy, leading to the development of biodegradable and non-toxic products (Purwatiningsih et al., 2012). Several studies reported the antifungal activity of the EO of *L. petersonii* on *Candida albicans* and several *Aspergillus* species (Hood et al., 2010; Kim and Park, 2012) and its antibacterial efficacy (Lis-Balchin et al., 1996; Demuner et al., 2011; Bugarin et al., 2014; Van Vuuren et al., 2014).

*Eucalyptus gunnii* Hook. F. (cinder gum) is a medium-sized tree, endemic to Tasmania (Brooker and Kleinig, 1996), and widely cultivated in France, the United Kingdom, Ireland, and Italy, mainly for the market of cut foliage (Forrest, 2002). Although *Eucalyptus* EO has a widely ascertained spectrum of biological activities including anti-microbial, fungicidal, insecticidal/insect repellent, herbicidal, acaricidal, and nematicidal (Batish et al., 2008), few papers have reported the chemical composition or properties of *E. gunnii* EO (Lucia et al., 2008; Elaissi et al., 2010; Bugarin et al., 2014). It has a weak anti-inflammatory effect (Dhakad et al., 2018) and major antibacterial properties against *Pseudomonas aeruginosa* when compared to EOs from other species of *Eucalyptus* genus (Elaissi et al., 2010; Salehi et al., 2019). To date, the phytotoxic activity, as well as the anti-biofilm properties, of these two EOs against several bacterial strains such as *Staphylococcus aureus*, *P. aeruginosa*, *Listeria monocytogenes*, and *Pectobacterium carotovorum* have not been investigated.

The persistent use of synthetic herbicides may impede sustainable agricultural production, causing ecological and environmental concerns such as increased resistance of weeds, environmental pollution, and health hazards (Rolim de Almeida et al., 2010). EOs are valuable sources of lead molecules in agriculture for weed and pest management. It is thus pertinent to explore and characterize the phytotoxic properties of aromatic plants.

In addition, biofilm formation and bacterial antibiotic resistance constitute problems of particular bearing for human health. For this reason, research aimed at identifying new antimicrobial compounds from natural sources such as medicinal plants are very topical.

Biofilm formation by *S. aureus* and *P. aeruginosa* is important when studying infections of the upper airways, because it perpetuates antigen presentation, leading to chronic inflammation and complicating clinical treatments (Camporese, 2013). Similar problems are caused by the uropathogenic *Escherichia coli*, whose biofilms are difficult to eradicate from the surface of hospital catheters (Bernal-Mercado et al., 2019), and *L. monocytogenes*, which form biofilms on food products (Djordjevic et al., 2002). Finally, *P. carotovorum* biofilm causes soft rot in food crops due the release of exoenzymes and increases bacterial resistance during plant disinfection (Gutierrez-Pacheco et al., 2018). *P. carotovorum* subsp. *brasiliense* was reported to form biofilm-like aggregates inside xylem vessels of tomato and potato plants (Kubheka et al., 2013). From this point of view, several EOs have already been tested (Hosseini Nezhad et al., 2012). Biofilm cells profoundly differ from planktonic cells of the same species because of phenotypic and metabolic changes that regulate some cell events, such as sporulation, starvation survival, adhesion, rough-smooth phase variations, etc. This also led to differences in the susceptibility of planktonic and biofilm cells to antimicrobial agents because adhesion-dependent phenotypic changes may be important as diffusion barriers or as growth rate-dependent changes in this important phenomenon. The metabolic changes can also make these cells inherently resistant to a particular antibiotic agent, virtually developed against specific targets in planktonic cells.

The present study was carried out to characterize the micromorphological features of *L. petersonii* and *E. gunnii* leaves by scanning electron microscopy (SEM) and to investigate the chemical composition of their EOs, as well as their antimicrobial and phytotoxic activities. The antibacterial activity was evaluated against some Gram-positive (*S. aureus* and *L. monocytogenes*) and Gram-negative (*E. coli* and *P. aeruginosa*) pathogenic strains and against the phytopathogen, *P. carotovorum*. The potential *in vitro* phytotoxicity was evaluated against germination and radical elongation of *Raphanus sativus* L. (radish), *Lactuca sativa* L.(lettuce), *Lepidium sativum* L. (garden cress), *Solanum lycopersicum* L. (tomato), *Lolium multiflorum* Lam. (Italian ryegrass), and *Portulaca oleracea* L.(purslane) seeds.

## Materials and Methods

### Plant Material

Branches with leaves of *L. petersonii*, belonging to the chemical variety “B CT Australian Rose,” were obtained from plantations situated in the Byron Bay hinterland, Northern New South Wales, Australia. Branches bearing young rounded leaves of *E. gunnii* were collected from pruning material of trees growing on a private land in Sardinia (Italy). Taxonomic identification of plant samples was carried out by MV for *E. gunnii* and GT for *L. petersonii*.

### Chemicals and Reagents

Ethanol-FineFix working solution was obtained from Milestone s.r.l., Bergamo, Italy. The bacterial culture medium, PBS, DMSO, tetracycline, ciprofloxacin, and MTT were supplied by Sigma, Milano, Italy. All other reagents were of analytical grade.

### Scanning Electron Microscopy

Samples of leaves (1–1.5 cm^2^) were fixed in a 70% ethanol-FineFix working solution (Milestone s.r.l., Bergamo, Italy) for 24 h at 4°C, and then gradually dehydrated by ethanol series (Chieco et al., 2012). Subsequently, samples were processed in a critical point dryer (CPD processor, K850 2M Strumenti s.r.l., Rome, Italy), mounted on aluminum stubs using glued carbon tabs, and sputter-coated with gold. Specimens were analyzed and photographed using an FE-SEM (Supra VP-40; Zeiss, Oberkochen, Germany) at an accelerating voltage of 20 kV.

### Isolation and Analysis of Essential Oils

#### Isolation of Essential Oils

Fresh plant materials were subjected to steam distillation until no significant increase in the volume of the collected EO was observed (3 h).

The EO yield (*w/v*, %) was calculated according to the following equation:

(1) $$Yield\left(\%\right)=\frac{W_{o}\times 100}{V_{E\,O}}$$

where W_0_ is the plant material weight distillated and V_EO_ is the EO volume obtained.

Essential oils were dried on Na_2_SO_4_ and stored in a dark-sealed vial with nitrogen headspace until analysis.

#### GC-FID Analysis

Analytical GC was performed on a Perkin-Elmer Sigma-115 gas chromatograph equipped with an FID and a data handling processor. The separation was achieved using a HP-5 MS fused-silica capillary column (30 m × 0.25 mm i.d., 0.25 μm film thickness). Column temperature: 40°C, with 5 min initial hold, and then to 270°C at 2°C/min, 270°C (20 min); injection mode splitless (1 μL of a 1:1000 *n*-hexane solution). Injector and detector temperatures were 250 and 290°C, respectively. Analysis was also run by using a fused silica HP Innowax polyethylene glycol capillary column (50 m × 0.20 mm i.d., 0.25 μm film thickness). In both cases, helium was used as a carrier gas (1.0 mL/min).

#### GC/MS Analysis and Identification of Single Constituents

Analyses were performed on an Agilent 6850 Ser. II apparatus, fitted with a fused silica DB-5 capillary column (30 m × 0.25 mm i.d., 0.33 μm film thickness), coupled to an Agilent Mass Selective Detector MSD 5973, ionization energy voltage 70 eV, electron multiplier voltage energy 2000 V. Mass spectra were acquired in the range 40–500 amu, scan time 5 scans/s. Gas chromatographic conditions were as reported above, with a transfer line temperature of 295°C. Most constituents were identified by comparison of their Kovats retention indices (Ri) [calculated in relation to retention time of *n*-alkanes (C_10_–C_35_)], with either those of the literature (Jennings and Shibamoto, 1980; Davies, 1990; Adams, 2007; Goodner, 2008), by accurate analysis of mass spectra on both columns and by their comparison with those of authentic compounds available in our laboratories by means of NIST 02 and Wiley 275 libraries (Wiley, 1998). The components’ relative concentrations were obtained by peak area normalization.

### Antibacterial Activity

#### Microorganisms and Culture Conditions

*Listeria monocytogenes* (ATCC 7644) and *P. aeruginosa* (ATCC 50071) were purchased from American Type Culture Collection (ATCC), MD, United States. *S. aureus*, *E. coli* (DSM 8579), and *Pectobacterium carovotorum* (DSM 102074) were purchased from Deutsche Sammlung von Mikroorganismen (DSM) collection, Braunschweig, Germany.

Bacteria were grown in Luria–Bertani (LB) broth (Sigma, Milan, Italy) for 18 h at 37°C and 80 r/min (Corning LSE, Pisa, Italy). *P. carovotorum* was grown at 28°C and 80 r/min.

#### Determination of the Antibacterial Susceptibility by Agar Diffusion Assay

Different concentrations of Eos, ranging from 0.01 to 2.0 μg/mL, diluted in sterile DMSO (Sigma–Aldrich Italy, Milan, Italy) were spotted onto the plates previously inoculated with 0.5 McFarland (1.5 × 10^7^ cells/mL) bacterial suspension (Densitometer cell density turbidity 0.3–15.0 McFarland, CAMLAB, Cambridge, United Kingdom) (Fratianni et al., 2016). After 10 min in sterile conditions, the plates were incubated for 24 h at 37 or 28°C, depending on the strain. The diameter of the clear zone shown on plates (inhibition zone) was accurately measured by using an Extra steel Caliper mod 0289, mm/inch reading scale, precision 0.05 mm (Mario De Maio, Milan, Italy). Sterile 1% DMSO and tetracycline (7 μg/mL, Sigma–Aldrich Italy, Milan, Italy) were used as negative and positive controls, respectively. The experiments were performed in triplicate and results expressed as mean ± SD.

#### Minimal Inhibitory Concentration (MIC)

The MIC values were calculated through the application of the resazurin microtiter-plate assay (Sarker et al., 2007). The EOs were dissolved in sterile DMSO. Twofold serial dilutions were prepared to obtain 50 μL of the EOs in serially descending concentrations in each well. Thirty-five μL of 3.3 × strength iso-sensitized broth and 5 μL of resazurin, used as indicator solution, were added to reach a final volume/well of 240 μL with several volumes of sterile Muller–Hinton broth (Sigma–Aldrich, Milan, Italy) previously set. Finally, 10 μL of bacterial suspension was added to each well to reach a concentration of about 5 × 10^5^ cfu/mL. Sterile DMSO and ciprofloxacin (Sigma–Aldrich Italy, Milan, Italy, 1 mg/mL in DMSO) were used as negative and positive controls, respectively. Multiwell plates were prepared in triplicate and incubated at 37°C for 24 h. The lowest concentration at which a color change occurred (from dark purple to colorless) revealed the MIC value.

#### Biofilm Inhibitory Activity

The effect of the EOs on bacterial adhesion ability was assessed in flat-bottomed 96-well microtiter plates according to the method of O’Toole and Kolter (1998), using EO concentrations ranging from 0.01 to 2.0 μg/mL (corresponding to volumes ranging from 1 to 20 μL, respectively).

In each well, the overnight bacterial cultures were adjusted to 0.5 McFarland (1.5 × 10^7^ cells/mL, Densitometer cell density turbidity 0.3–15.0 McFarland, CAMLAB, Cambridge, United Kingdom) with fresh culture broth. Then, 10 μL of the diluted cultures was distributed in each well, and different volumes of the extracts and Muller–Hinton broth were added, to reach a final volume of 250 μL/well. Microplates were completely covered with parafilm, to avoid the evaporation of samples with relative loss of volume and incubated for 48 h at different temperatures (depending on the strain). Planktonic cells were removed and the attached cells were gently twice washed with sterile physiological saline. After that, 200 μL of methanol was added to each well, retaining it for 15 min to fix the sessile cells. Methanol was then discarded, and each plate was left until complete dryness of samples. The staining of the adhered cells was obtained by adding 200 μL of 2% *w/v* crystal violet solution to each well that was left for 20 min. Wells were gently washed with sterile physiological solution and left to dry. Two hundred microliters of glacial acetic acid 20% *w/v* were added to allow the release of the bound dye. The absorbance was measured at OD = 540 nm (Varian Cary Spectrophotometer model 50 MPR, Cernusco sul Naviglio, Italy). The percent value of biofilm inhibition was calculated with respect to control (cells grown without the presence of the EOs). Triplicate tests were done, and the average results were taken for reproducibility.

#### Metabolic Activity of Biofilm Cells

The effect of different concentrations of Eos, ranging from 0.01 to 2.0 μg/mL on the metabolic activity of biofilm cells, was evaluated through the MTT colorimetric method according to Kairo et al. (1999) and Fratianni et al. (2019), using 96-well microtiter plates.

The overnight bacterial cultures were adjusted to 0.5 McFarland and treated as described in Section “Biofilm Inhibitory Activity.”

After 48 h incubation, bacterial suspension was removed and 150 μL of PBS and 30 μL of 0.3% MTT (Sigma, Milan, Italy) were added, keeping microplates at 37°C. After 2 h, the MTT solution was removed, two washing steps were performed with 200 μL of sterile physiological solution, and 200 μL of DMSO was added to allow the dissolution of the formazan crystals, which were measured after 2 h at OD = 570 nm (Varian Cary Spectrophotometer model 50 MPR, Cernusco sul Naviglio, Italy). Triplicate tests were carried out and the average results were taken for reproducibility.

### Phytotoxic Activity

The phytotoxic activity was evaluated on germination and radical elongation of *R. sativus* L. (radish), *L. sativa* L. (lettuce), *L. sativum* L. (garden cress), *S. lycopersicum* L. (tomato), *L. multiflorum* Lam. (Italian ryegrass), and *P. oleracea* L. (purslane). These seeds are usually used in phytotoxicity assays because they easily germinate and are well known from a histological point of view. Radish, lettuce, garden cress, and tomato seeds were purchased from the Blumen Group s.r.l. (Emilia Romagna, Italy), Italian ryegrass seeds were purchased from Fratelli Ingegnoli Spa (Milano, Italy), and purslane seeds from W. Legutko s.r.l. (Jutrosin, Poland). The seeds were surface sterilized in 95% ethanol for 15 s and sown in Petri dishes (Ø = 90 mm), containing three layers of Whatman filter paper, and impregnated with 7 mL of distilled water (control) or 7 mL of the tested solution of EO. The germination conditions were 20 ± 1°C, with a natural photoperiod. The EOs, dissolved into water-acetone mixture (99.5:0.5), were assayed at the doses of 100, 10, 1, and 0.1 μg/mL. Controls performed with water–acetone mixture alone showed no differences in comparison to controls in water alone. Seed germination was observed directly in Petri dishes every 24 h. A seed was considered germinated when the protrusion of the root became evident (Bewley, 1997). After 120 h (on the fifth day), the effects on radicle elongation were measured in cm. Each determination was repeated three times, using Petri dishes containing 10 seeds each. Data were expressed as mean ± SD for both germination and radical elongation.

### Statistical Analysis

All experiments were carried out in triplicate. Data from each experiment were statistically analyzed using GraphPad Prism 6.0 software (GraphPad Software Inc., San Diego, CA, United States) followed by comparison of means (two-way ANOVA) using Dunnett’s multiple comparisons test, at the significance level of *p* < 0.05.

## Results and Discussion

### Micromorphological Characterization

The leaf anatomical features of *L. petersonii* and *E. gunnii* have been scarcely investigated, although leaves are the main section of the plant from which EOs are extracted. Concerning *E. gunnii*, most recent studies have been focused on the epicuticular waxes of the leaf surface, considering their morphology, composition, function, and biosynthesis, and also regarding the modulatory effects of different stress factors (Shepherd and Griffiths, 2006) and regeneration processes after removal (Huth et al., 2018). A brief anatomical description of *L. petersonii* leaf anatomy has been reported by Johnson (1980) in his comprehensive revision of the genus *Leptospermum*, taking into account 40 different species.

**FIGURE 1:**
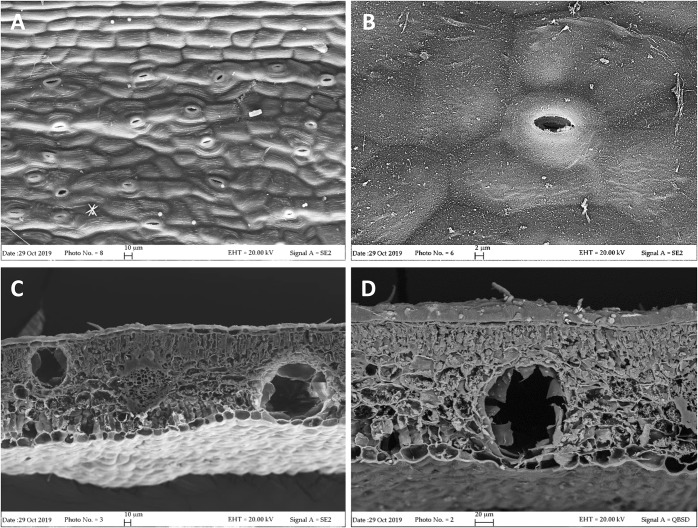
SEM micrographs of *L. petersonii* leaves. **(A,B)** Epidermal surface showing subpolygonal striate cells and scattered paracytic stomata. **(C)** Leaf transversal section showing two secretory cavities, located on each side of the rib, one close to the upper epidermis and the other one to the lower epidermis. **(D)** Higher magnification of an oil cavity located close to the lower epidermis of the leaf.

**FIGURE 2:**
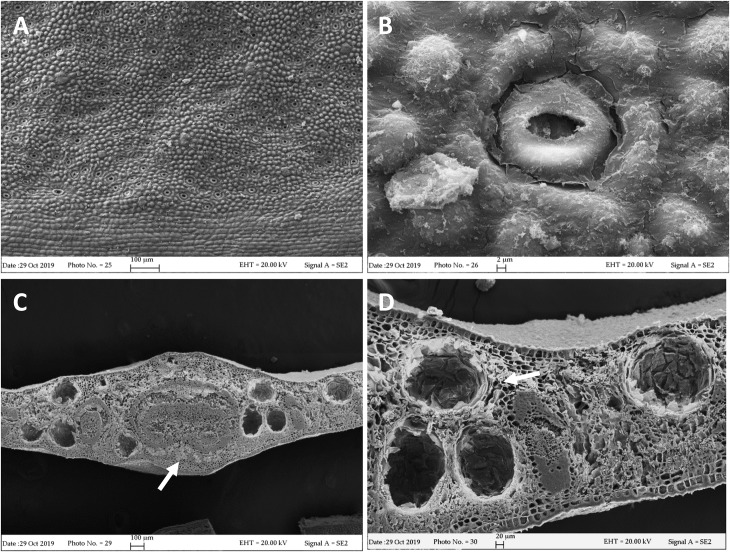
SEM micrographs of *E. gunnii* leaf. **(A,B)** Epidermal surface showing papillose cells and many anomocytic stomata. **(C)** Leaf trasversal section with secretory cavities scattered throughout the mesophyll, around the midrib. **(C,D)** Prismatic crystals and druses are visible near and around the secretory cavities and the rib (arrows).

Our FE-SEM investigation has highlighted many typical xeromorphic adaptations against water loss, such as sunkenstomata, thick cuticles, and a waxy epidermis in the leaves of *L. petersonii* (Figures 1A,B), and even more distinctly in those of *E. gunnii* (Figures 2A,B). The cuticle on the leaf epidermal surfaces of *E. gunnii* is smooth or slightly striated and shows cuticular papillae and epicuticular waxes shaped as small granules (Figure 2B). These features appear similar to those reported by Migacz et al. (2018) for *Eucalyptus dunni* Maiden. The mesophyll was dorsiventral in *L. petersonii* (Figures 1C,D), whereas it appeared isobilateral in *E. gunnii* (Figures 2C,D). The oil glands of both species have previously been found to develop by schizogony of cell walls (Carr and Carr, 1970; Johnson, 1980). In our observations, these schizogenous cavities appeared scattered in the mesophyll, especially in the sub-epidermal region, on both leaf sides (Figures 1, 2C,D). In transversal sections of *E. gunnii* leaves, many prismatic crystals and druses of calcium oxalate were also found, mainly around the ribs and near the oil glands (Figures 2C,D, arrows). On the contrary, prismatic crystals were absent on the leaf epidermal surface, being one of the diagnostic features useful to differentiate *Eucalyptus* species (Migacz et al., 2018).

The leaf morphological features detected in the examined species can be useful for their identification or in the quality control of herbal material used for EO extraction, to avoid accidental or intentional adulteration.

### Essential Oils Yield and Chemical Composition

#### Essential Oil Yields

Steam distillation of the chemical variety “B CT Australian Rose” of *L. petersonii* and *E. gunnii* branchlets and leaves furnished EOs in 0.5 and 2% yields, respectively. The yield of our sample of *L. petersonii* was higher than other species of genus Leptospermum. In fact, Windsor and Brooks (2012) in their study observed a yield of 0.01–0.06% for *Leptospermum laevigatum*, 0.06–0.2% *Leptospermum speciosum*, and 0.2–0.4% for *Leptospermum trinervium*. Van Vuuren et al. (2014) demonstrated that for *L. petersonii* annual EO yields ranged from 0.4% (in February) and 1.8% (in March). Moreover, Demuner et al. (2011) showed that flower *L. petersonii* (0.5%) EO was lower than that found for the leaves (3.7%) in the same period.

Also, for genus *Eucalyptus*, the yields varied according to the species. In fact, Elaissi et al. (2010) analyzed 13 species from Tunisia and demonstrated yields ranging from 0.5%, for *E. gunnii* to 3.9% for *Eucalyptus cinerea*. Moreover, comparing different *Eucalyptus* species, Lucia et al. (2008) showed a significant relationship between total EO yields and 1.8-cineole concentration.

This shows that there are different factors that can lead to quantitative and qualitative differences in the EOs such as soil, season, temperature, geographic origin, and ecological role of the plant organs that produce EOs (Demuner et al., 2011).

#### Chemical Composition of Essential Oils

Essential oil compositions with retention indices and area percentages for each compound are reported in Tables 1, 2.

**TABLE 1:** Chemical composition of *L. petersonii* “variety B CT Australian Rose” essential oil.

N.	Compound	%	KI^a^	KI^b^	Identification^c^
1	(E)-2-Octene	t	744	818	1,2
2	3-Methyl-2-buten-1-ol	t	758	778	1,2
3	(Z)-3-Hexen-1-ol	0.2	807	851	1,2
4	Heptanal	t	821	899	1,2
5	α-Pinene	5.7	857	939	1,2,3
6	Camphene	0.1	872	954	1,2,3
7	δ-2-Carene	0.5	896	1002	1,2,3
8	α-Fenchene	2.2	915	952	1,2
9	α-Phellandrene	0.4	923	1002	1,2
10	*iso*-Sylvestrene	2.3	935	1008	1,2
11	*p*-Cimene	5.6	944	1024	1,2,3
12	Limonene	2.9	947	1029	1,2,3
13	1,8-Cineole	0.5	948	1031	1,2,3
14	(Z)-β-ocimene	0.3	958	1037	1,2,3
15	(E)-β-ocimene	5.1	968	1050	1,2,3
16	γ-Terpinene	12.4	978	1059	1,2,3
17	Terpinolene	9.3	1000	1088	1,2
18	Methylbenzoate	0.1	1006	1090	1,2
19	1-Terpineol	0.1	1011	1133	1,2
20	Linalool	5.1	1018	1096	1,2,3
21	1,3,8-*p*-Menthatriene	0.1	1020	1110	1,2
29	Geraniol	9.5		1252	1,2
30	3- Thujanol acetate	0.1	1196	1295	1,2
31	Thymol	t	1210	1290	1,2,3
32	Perillaaldehyde	0.1	1217	1271	1,2
33	γ-Elemene	0.7	1224	1338	1,2
34	α-Cubebene	t	1236	1348	1,2
35	Geraniol	t	1241	1252	1,2,3
36	*cis*-Myrtanol	t	1246	1253	1,2
37	Eugenol	0.6	1257	1359	1,2,3
38	α-Ylangene	0.1	1260	1375	1,2
39	Isoledene	t	1263	1376	1,2
40	Geranyl-acetate	31.4	1286	1384	1,2,3
41	*trans*-Muurola-3,5-diene	0.2	1307	1453	1,2
42	α-*neo*-Clovene	0.9	1316	1454	1,2
43	α-Humulene	0.1	1330	1454	1,2,3
44	α-Patchoulene	0.2	1337	1456	1,2
45	*allo*-Aromadendrene	0.3	1354	1460	1,2,3
46	9-*epi*-(E)-Caryophyllene	0.1	1357	1466	1,2
47	β-Acoradiene	0.1	1367	1470	1,2
48	Cumacrene	0.7	1372	1472	1,2
49	Dauca-5,8-diene	0.1	1378	1472	1,2
50	α-Neocallitropsene	t	1383	1476	1,2
51	Dehydro-aromadendrane	0.1	1388	1462	1,2
52	*cis*-Cadina-1(6),4-diene	0.1	1389	1463	1,2
53	*trans*-Cadina-1(6),4-diene	0.4	1394	1476	1,2
54	β-Chamigrene	0.1	1422	1477	1,2
55	β-Selinene	t	1435	1490	1,2
56	Viridiflorene	0.6	1453	1496	1,2
57	Modhephen-8-β-ol	t	1471	1513	1,2
58	Cubenol	t	1496	1646	1,2
59	Rosifoliol	0.1	1510	1600	1,2
60	10-epi-γ-Eudesmol	0.1	1514	1623	1,2
61	γ-Eudesmol	t	1516	1632	1,2
62	β-Atlantol	t	1523	1608	1,2
63	Cedr-8-(15)-en-9-α-ol	t	1579	1651	1,2
64	Benzylbenzoate	t	1622	1760	1,2
	Total	98.9			
	Monoterpenehydrocarbons	46.4			
	Oxygenatedmonoterpenes	47.2			
	Sesquiterpenehydrocarbons	4.8			
	Oxygenatedsesquiterpenes	0.2			
	Others	0.3			

*^*a*^Linear retention index on a HP-5MS column; ^*b*^Linear retention index on a HP Innowax column; ^*c*^Identification method: 1 = linear retention index; 2 = identification based on the comparison of mass spectra; 3 = Co-injection withstandard compounds; t = traces, less than 0.1%.*

**TABLE 2:** Chemical composition of *E. gunnii* essential oil.

N.	Compound	%	KI^a^	KI^b^	Identification^c^
1	α-Pinene	0.3	942	939	1,2,3
2	Camphene	6.3	945	954	1,2,3
3	1,8 Cineole	33.0	946	1026	1,2,3
4	δ-2-Carene	0.7	997	1002	1,2,3
5	*m*-Cymenene	0.1	999	1085	1,2
6	Dehydro-linalool	2.3	1045	1090	1,2
7	1-*p*-Menthene	0.3	1073	1026	1,2
8	γ-Terpinene	0.3	1075	1059	1,2,3
9	*cis*-Sabinene hydrate	0.9	1083	1070	1,2
10	1,3,8-*p*-Menthatriene	1.0	1095	1110	1,2
11	Terpinolene	5.9	1097	1088	1,2,3
12	*trans*-*p*-Mentha-2,8-dien-1-ol	0.8	1129	1122	1,2
13	Silphinene	0.5	1224	1347	1,2
14	*trans*-Sabinene hydrate acetate	15.0	1238	1256	1,2
15	Presilphiperfol-7-ene	0.7	1292	1336	1,2
16	Longicyclene	9.1	1313	1374	1,2,3
17	(Z)-Caryophyllene	0.3	1360	1408	1,2,3
18	α-Gurjunene	1.8	1370	1409	1,2
19	Aromadendrene	2.7	1429	1441	1,2,3
20	Spathulenol	0.6	1447	1578	1,2
21	Globulol	10.3	1452	1590	1,2,3
22	Viridiflorol	2.6	1460	1592	1,2,3
23	Cubeban-11-ol	1.1	1462	1595	1,2
24	Rosifoliol	1.4	1470	1600	1,2
	Total	98.0			
	Monoterpene hydrocarbons	15.9			
	Oxygenated monoterpenes	51.0			
	Sesquiterpenes hydrocarbons	15.1			
	Oxygenated sesquiterpenes	16.0			

*^*a*^Linear retention index on a HP-5MS column; ^*b*^Linear retention index on a HP Innowax column; ^*c*^Identification method: 1 = linear retention index; 2 = identification based on the comparison of mass spectra; 3 = co-injection with standard compounds.*

Sixty-four compounds were identified for *L. petersonii* EO, accounting for 98.9% of the total EO. In particular, the main constituents are geranyl acetate (31.4%), geraniol (9.5%), linalool (5.1%) as oxygenated monoterpenes and γ-terpinene (12.4%), terpinolene (9.3%), α-pinene (5.7%), p-cimene (5.6%), and (E)-β-Ocimene (5.1%) among monoterpenes hydrocarbons.

In the EO from *E. gunnii.* 24 compounds were identified, accounting for 97.9% of the total EO. The main compounds were: oxygenated monoterpenes with 1,8-cineole (33.0%), trans-sabinene hydrate acetate (15.0%), and globulol (10.3%).

Other minor components were α-fenchene (2.2%), iso-sylvestrene (2.3%), and limonene (2.9%) into *L. petersonii* EO, and longicyclene (9.1%), terpinolene (5.9%), and camphene (6.3%), aromadendrene (2.7%), viridiflorol (2.6%), and dehydro-linalool (2.3%) into *E. gunnii* EO.

Seven constituents were present in both EOs but in different amounts. The percentages of α-pinene (5.7%) and γ-terpinene (12.4%) in *L. petersonii* EO was higher than those in *E. gunnii* (0.3% for both compounds); instead, camphene (6.3%), 1,8 cineole (33.0%), and rosifoliol (1.4%) were present more in *E. gunnii* EO than in *L. petersoniii* (0.1, 0.5, and 0.1%, respectively). δ-2-carene and terpinolene were found in similar amounts in both oils, respectively, 0.7 and 5.9% in *E. gunnii* and 0.5 and 9.3% in *L. petersonii* EO.

The composition of the EO of *L. petersonii* chemical variety “B CT Australian Rose” of this study was partially in agreement with previous data. In fact, structural analogs of geraniol, such as geranial (34.7–29.9%) and neral (19.7–23.5%), have been reported as the main constituents of *L. petersonii* EO. On the other hand, constituents reported in high amounts in other studies, such as citronellal (33.9–11.4%) or citronellol (17.5%), were absent in our sample (Demuner et al., 2011; Kim and Park, 2012; Van Vuuren et al., 2014).

The different composition of the EOs confirmed the existence of several chemotypes in *L. petersonii*, as reported by Brophy et al. (2000). The most common chemical variety with a pleasant odor is the “A” type, which consists mainly of aldehydes such as neral, geranial, citronellal, and monoterpene hydrocarbons (γ-terpinene, α-pinene, *p*-cymene). On the contrary, the chemical variety “B,” with a rose-like odor, is quite rare and contains geraniol and geranyl acetate as main constituents, followed by γ-terpinene and terpinolene (Brophy et al., 2000).

There have been no large studies on the rare chemical variety “B”: the sample analyzed in this study was evidently derived from *L. petersonii* chemical variety “B.” In fact, our results agree with Brophy et al. (2000), who reported the chemical composition of three EOs belonging to this variety, with geranyl acetate (ranging from 21 to 38%) and geraniol (ranging from 21 to 29%) as the main components.

The chemical composition of the analyzed *E. gunnii* EO is quite similar to data reported in literature. 1,8 Cineole was confirmed as the main component, as described in previous studies, with a percentage ranging from 17.9 to 67.8% (Lucia et al., 2008; Bugarin et al., 2014). Conversely, spathulenol (16.5%) was reported as the major component of *E. gunnii* EO from Tunisia (Elaissi et al., 2011) and it was identified also in *E. gunnii* EO from Argentina with a percentage of 12.3% (Lucia et al., 2008). This compound was present in a very low amount (0.6%) in our EO. Moreover, viridiflorol and globulol were present in a high percentage in Tunisian EO (11.5 and 12.5%, respectively) (Elaissi et al., 2011). These compounds were also present in our sample, in particular globulolin in a similar quantity (10.3%), while viridiflorol was present in a lower amount (0.6%).

### Antibacterial Activity

**FIGURE 3:**
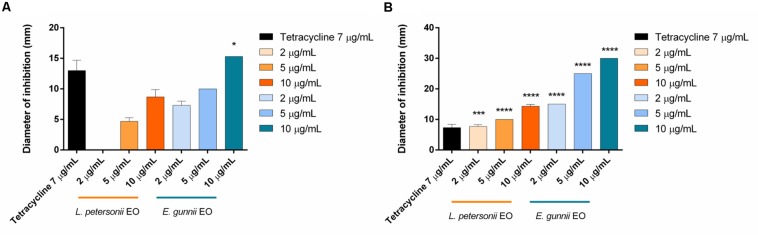
Antibacterial activity of *L. petersonii* and *E. gunnii* EOs and of tetracycline against the Gram-positive *L. monocytogenes* **(A)** and *S. aureus* **(B)**. Results are the mean of three experiments. Dunnett’s test *vs* tetracycline 7 μg/mL (*p < 0.05; ***p < 0.001; ****p < 0.0001).

The antibacterial activity of the EOs was evaluated against Gram-positive and Gram-negative pathogenic strains, through the inhibition zone test and the determination of the MIC. Results are shown in Figure 3 and in Table 3, respectively.

**TABLE 3:** MIC (μg/mL) of the EOs of *L. petersonii* and *E. gunnii*.

	MIC (μg/mL)	
Microorganism	*L. petersonii*	*E. gunnii*
*E. coli*	2.0 (±0.20)	1.0 (±0.00)
*L. monocytogenes*	5.0 (±0.50)	1.0 (±0.00)
*P. carotovorum*	5.0 (±0.50)	2.0 (±0.20)
*P. aeruginosa*	1.0 (±0.00)	1.0 (±0.00)
*S. aureus*	1.0 (±0.00)	0.5 (±0.00)

*Results are the mean of three experiments ± SD.*

The behavior of the two EOs against Gram-positive (*S. aureus* and *L. monocytogenes*) and Gram-negative (*E. coli*, *P. aeruginosa*, and *P. carotovorum*) strains was completely different. *E. gunnii* EO showed the best antibacterial activity against Gram-positive strains. Indeed, at highest concentration (10 μg/mL), the inhibition zone *vs S. aureus* and *L. monocytegenes* was 2 and 2.5 times greater than those obtained using the same concentration of *L. petersonii* EO (Figure 3). Moreover, the highest concentration of *E. gunnii* EO was much more effective than the reference antibiotic against *S. aureus* and even more so against *L. monocytogenes.*

The different behavior showed by the two EOs at the highest concentration could be ascribable to their different phytochemical composition. The presence of 1.8-cineole (33%) and trans-sabinene hydrate-acetate (15%) in *E. gunnii* EO probably contributes to the strong antibacterial activity of this EO, according to Nazzaro et al. (2013). Indeed, 1.8-cineole is a well-known antibacterial agent with proven activity against all the microorganisms used in our experiments (Caputo et al., 2017). Furthermore, γ-terpinene and trans-sabinene hydrate-acetate could enhance the *E. gunnii* EO ability to block the bacterial growth (Nazzaro et al., 2013), probably due to a synergistic mechanism (Dorman and Deans, 2000). Finally, globulol (10.3%), which was identified in other *Eucalyptus* EOs, also possesses antibacterial activity (Sebei et al., 2015).

On the contrary, there is no positive correlation between the high amount of geranyl-acetate in *L. petersonii* EO and antibacterial efficacy *vs L. monocytogenes* (Lis-Balchin and Deans, 1997).

**FIGURE 4:**
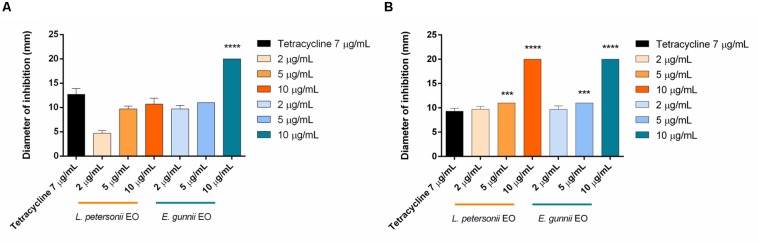
Antibacterial activity of *L. petersonii* and *E. gunnii* EOs and of tetracycline against the Gram-negative *E. coli* **(A)** and *P. aeruginosa* **(B)**. Results are the mean of three experiments. Dunnett’s test *vs* tetracycline 7 μg/mL (***p < 0.001; ****p < 0.0001).

Considering the Gram-negative microorganisms tested, the results obtained would allow differentiating two subgroups for resistance/sensitivity to the *L. petersonii* and *E. gunnii* EOs, a first sub-group represented by *E. coli* and *P. aeruginosa*, and a second one by *P. carotovorum*. *E. gunnii* EO showed good activity against *E. coli*, although it was weaker than that exerted on other *E. coli* strains (Bugarin et al., 2014). It is well known that the resistance/sensitivity to an antimicrobial agent may vary among different strains of the same species (Del Monte et al., 2015). However, we should stress that the inhibition zone induced by *E. gunnii* EO was almost double than tetracycline (Figure 4A). *E. gunnii* EO also showed strong activity against *P. aeruginosa*, with an inhibition diameter twice with respect to tetracycline (Figure 4B). Thus, this EO can represent a product with a wide potential against pathogenic species such as *E. coli*, *P. aeruginosa*, *L. monocytogenes*, and *S. aureus*, resulting in particular interest due to the increased number of microbial species showing resistance to antibiotic drugs (Heras et al., 2015; Hartmann et al., 2019).

*Leptospermum petersonii* EO was usually less effective in comparison with *E. gunnii* EO, as indicated by the MIC values (Table 3). Moreover, despite exercising an inhibitory activity against *E coli* at all the concentrations used, its efficacy (width of the inhibition zone) was always less than tetracycline (Figure 4A). On the contrary, it was more effective *vs Pseudomonas*, with inhibition zones of about 20 mm, practically double with respect to tetracycline. Indeed, in this case, the inhibiting force was similar to that exerted by the *E. gunnii* EO (Figure 4B). The effectiveness shown by *L. petersonii* EO against *P. aeruginosa* and *S. aureus* (Figures 3, 4B) is in accordance with Demuner et al. (2011), which demonstrated a strong efficacy of *Leptospermum* spp. EOs against these bacteria. The composition of *L. petersonii* EO from Brazil and South Africa, with considerable amounts of citronellal, geranial, and neral, may confer forceful antimicrobial activity (Van Vuuren et al., 2014).

An MIC test confirmed the more powerful activity of *E. gunnii* EO (Table 3), with MIC values which ranged between 0.5 and 2.0 μg/mL (against *S. aureus* and *P. carotovorum*, respectively). The activity of *E. gunnii* EO was generally stronger than *L. petersonii* EO, whose maximum MIC value reached even 5 μg/mL. MIC values of *L. petersonii* EO were usually higher against *L. monocytogenes* (Table 3).

*Pectobacterium* (formerly *Erwinia*) *carotovorum* is a Gram-negative bacterium belonging to the Enterobacteriaceae family, which affects several crops such as potato, pineapple, maize, and African violet (Mehrsorosh et al., 2014). It causes soft rot and blackleg of potatoes and vegetables, as well as slime flux on many different tree species. The soft rot erwinias can be present on plant surfaces and in soil where they may penetrate within the plant via wound sites or through natural openings on the plant surface, e.g., lenticels.

**FIGURE 5:**
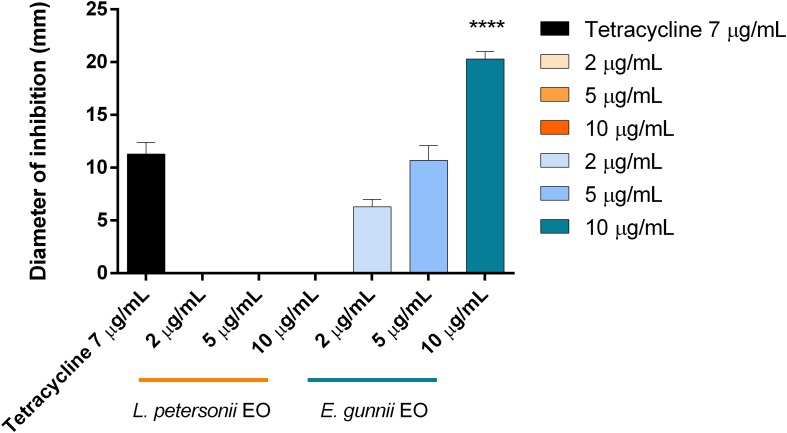
Antibacterial activity of *L. petersonii* and *E. gunnii* EOs and of tetracycline against *P. carotovorum.* Results are the mean of three experiments. Dunnett’s test *vs* tetracycline 7 μg/mL (****p < 0.0001).

In the antibacterial tests, we observed completely opposed behavior of the two EOs investigated. *E. gunnii* EO resulted active already at 2 μg/mL (Table 3), causing an inhibition zone of 7 mm (Figure 5). The highest concentration (10 μg/mL) showed an inhibition zone of 17 mm, almost double with respect to the diameter obtained with tetracycline (Figure 5).

To our knowledge, this is the first time that the antibacterial activity of *E. gunnii* EO against *P. carotovorum* has been evaluated. Such activity was stronger than those of EOs obtained from other species belonging to the *Eucalyptus* genus, such as *Eucalyptus caesia* Benth., *Eucalyptus camaldulensis* subsp. *obtusa* (Blakely Brooker and M. W. McDonald), and *Eucalyptus gomphocephala* A. Cunn. ex DC., and much more effective with respect to some Lamiaceae EOs (Mehrsorosh et al., 2014; Salem et al., 2015).

This high antibacterial activity against the Gram negative phytopathogen *P. carotovorum* makes this EO an ideal candidate to treat and prevent bacterial infections in crops; in addition, through the use of new species-specific technologies, such as the encapsulation of EO in mesoporous silica nanoparticles, this EO has high efficacy when applied to this and other phytopathogens (Cadena et al., 2018).

### Biofilm and Metabolic Activity of Biofilm Cells

*Eucalyptus gunnii* and *L. petersonii* EOs were tested on the above bacterial strains for inhibition of biofilm formation and metabolic activity. In our study, for the first time, biofilm inhibition by these two EOs (Table 4) was integrated with EO effects on biofilm metabolism (Table 5), thus increasing available information on their antibacterial power and potential applications.

**TABLE 4:** Capability of *E. gunnii* and *L. petersonii* EOs to inhibit the formation of biofilm.

Inhibition biofilm (% ± SD)								
	0.01 μg/mL	0.02 μg/mL	0.05 μg/mL	0.1 μg/mL	0.2 μg/mL	0.5 μg/mL	1.0 μg/mL	2.0 μg/mL
EC/EUC	0 (±0)	0 (±0)	0 (±0)	13.21 (±1.15)	64.24 (±1.67)	80.28 (±0.57)		
EC/LEPT	0 (±0)	0 (±0)	0 (±0)	0 (±0)	35.42 (±0.57)	54.17 (±0.57)	96.22 (±1.67)	
LM/EUC	0 (±0)	0 (±0)	0 (±0)	0 (±0)	4.88 (±0.57)	50.21 (±0.57)		
LM/LEPT	0 (±0)	0 (±0)	0 (±0)	35.91 (±1.67)	46.33 (±0.57)	53.11 (±0.57)	53.11 (±1.67)	78.41 (±0.57)
PC/EUC	0 (±0)	0 (±0)	0 (±0)	8.23 (±0.35)	90.44 (±1.67)	90.46 (±0.57)	93.12 (±1.15)	
PC/LEPT	0 (±0)	0 (±0)	0 (±0)	10.43 (±1.67)	20.43 (±1.67)	30.14 (±0.57)	47.62 (±1.67)	66.67 (±0.57)
PSA/EUC	0 (±0)	0 (±0)	0 (±0)	3.57 (±0.57)	26.23 (±1.67)	35.43 (±1.67)		
PSA/LEPT	0 (±0)	0 (±0)	0 (±0)	10.02 (±0.57)	20.1 (±0.57)	20.15 (±1.15)		
SA/EUC	10.37 (±1.67)	25.16 (±0.57)	46.23 (±0.57)	50.37 (±1.67)	60.17 (±1.67)			
SA/LEPT	14.78 (±1.67)	15.41 (±1.67)	18.79 (±1.15)	18.87 (±1.67)	49.88 (±1.67)	79.88 (±0.57)		

*Results are reported as percent of inhibition respect to the control (inhibition = 0). Results are the mean of three experiments ± SD. EC: *E. coli*; LM: *L. monocytogenes*; PC: *P. carotovorum*; PSA: *P. aeruginosa*; SA: *S. aureus*; EUC: *E. gunnii* EO; LEPT: *L. petersonii* EO.*

**TABLE 5:** Metabolic activity exhibited by the cells present within the bacterial biofilms in the presence of different concentrations of *E. gunnii* and *L. petersonii* EOs.

Metabolic activity of biofilm cells (% ± SD)								
	0.01 μg/mL	0.02 μg/mL	0.05 μg/mL	0.1 μg/mL	0.2 μg/mL	0.5 μg/mL	1.0 μg/mL	2.0 μg/mL
EC/EUC	100 (±0)	100 (±0)	100 (±0)	64.00 (±0.57)	60.81 (±1.67)	55.60 (±1.67)	52.92 (±1.15)	
EC/LEPT	100 (±0)	100 (±0)	100 (±0)	78.55 (±1.67)	63.91 (±1.15)	55.15 (±1.67)	48.24 (±0.57)	
LM/EUC	100 (±0)	100 (±0)	100 (±0)	100.0 (±0)	67.34 (±1.15)	60.35 (±1.67)		
LM/LEPT	100 (±0)	100 (±0)	100 (±0)	95.67 (±0.57)	95.06 (±0.57)	83.56 (±1.15)	50.64 (±1.67)	44.30 (±0.57)
PC/EUC	100 (±0)	100 (±0)	100 (±0)	40.80 (±1.67)	33.38 (±0.57)	32.18 (±0.57)	22.57 (±1.67)	
PC/LEPT	100 (±0)	100 (±0)	100 (±0)	43.62 (±0.57)	25.48 (±0.57)	23.32 (±1.67)	16.98 (±0.57)	16.09 (±0.57)
PSA/EUC	100 (±0)	100 (±0)	100 (±0)	94.35 (±0.57)	83.34 (±1.67)	77.74 (±1.67)		
PSA/LEPT	100 (±0)	100 (±0)	100 (±0)	93.39 (±0.57)	85.03 (±1.67)	83.55 (±1.67)		
SA/EUC	87.54 (±0)	72.10 (±0)	66.05 (±0.57)	64.32 (±1.67)	63.21 (±1.67)			
SA/LEPT	98.41 (±0.57)	94.38 (±0.57)	92.64 (±1.15)	91.54 (±1.67)	91.24 (±1.15)	90.89 (±0.57)		

*Results are expressed as metabolic activity percent, calculated through the MTT test respect to the control (100% of metabolic activity). Results are the mean of three experiments ± SD. EC: *E. coli*; LM: *L. monocytogenes*; PC: *P. carotovorum*; PSA: *P. aeruginosa*; SA: *S. aureus*; EUC: *E. gunnii* EO; LEPT: *L. petersonii* EO.*

The activity of *E. gunnii* EO against the uropathogen *E. coli* was of particular interest. Indeed, at a concentration of only 0.5 μg/mL, this EO causes a biofilm formation inhibition of 80.28%. These results were in accordance with other previous studies (Karpanen et al., 2008; Hendry et al., 2009), which have ascertained the antimicrobial activity of *Eucalyptus* EO *vs E. coli* and *Staphylococcus epidermidis* biofilm formation.

Moreover, *E. gunnii* EO decreased the metabolic activity of the *E. coli* biofilm cells to 55.6% with respect to the control, as observed by MTT assay (Table 5). This means that *E. gunnii* EO was capable not only of strongly inhibiting the *E. coli* biofilm formation but also to limiting all metabolic changes, which make cells more resistant to the antibacterial agents. *L. petersonii* EO, although less strong than the previous one, still shows inhibitory properties against *E. coli* biofilm formation (96.22%) and decreases the metabolic activity (48.24%) at 1.0 μg/mL (Tables 4, 5, respectively). The activity of *E. gunnii* and *L. petersoni* EOs against *E. coli* could be particularly important in clinical settings and patient management.

When EOs were tested to evaluate their ability to block *L. monocytogenes* biofilm formation, their behavior was different and *L. petersonii* EO showed, in general, the best antibacterial activity. Indeed, it inhibits the *L. monocytogenes* biofilm formation at 0.1 μg/mL, with an inhibition rate reaching 78.41% at 2 μg/mL. Moreover, it was able to influence the biofilm cells metabolism by decreasing their activity to 44.30% compared to the control (Table 5). On the contrary, *E. gunnii* EO was completely inactive until 0.1 μg/mL, showing an inhibition of biofilm formation of 4.8 and 50.21% at 0.2 and 0.5 μg/mL, respectively (Table 4). The latter concentration (0.5 μg/mL) showed a decrease of biofilm cells metabolism to 60.35% with respect to control (Table 5).

Essential oils investigated showed a weak inhibitory activity regarding both biofilm growth (35.43 and 20.15% by *E. gunnii* and *L. petersoni* EOs, respectively, Table 4) and metabolic activity of *P. aeruginosa* (77.74 and 83.55% by *E. gunnii* and *L. petersoni* EOs, respectively, Table 5).

This confirmed the weak antibacterial activity of *Eucalyptus* species against biofilm formation, as previously reported for *Eucalyptus globulus* Labill. EO (Sambyal et al., 2017), although its action could be enhanced through innovative biotechnological processes. For instance, nanoemulsion containing *E. globulus* EO showed antimicrobial and anti-biofilm activities against some Gram-negative bacteria, commonly found in immunosuppressed patients, such as *P. aeruginosa* and *C. albicans* (Quatrin et al., 2017).

The two EOs were particularly effective against *S. aureus* (Table 4), inhibiting biofilm formation by 10.37 and 14.78%, respectively, at a concentration of 0.01 μg/mL. The activity of *E. gunnii* EO on *S. aureus* was stronger than *L. petersonii* EO at a concentration above 0.01 μg/mL (Table 4). The inhibitory effect of *E. gunnii* EO against *S. aureus* confirmed the capability of the EOs recovered by species belonging to the *Eucalyptus* genus to block the biofilm formation by this methicillin resistant strain (Junka et al., 2019); its activity was more powerful than *E. globulus* EO (Merghni et al., 2018). The effectiveness shown by both EOs could be taken into consideration, once again, in patient management, mainly to avoid *S. aureus* infections that concern the central venous, dialysis (Gahlot et al., 2014), and urinary catheters (Murugan et al., 2016), as well as arterio-venous shunts (MacRae et al., 2016) and sutures (Henry-Stanley et al., 2010). Interestingly, *L. petersonii* EO at 0.5 μg/mL induced a strong inhibition of *S. aureus* biofilm formation (79.88%), while maintaining a high metabolic activity (90.89%) compared to the control.

*Eucalyptus gunnii* EO was particularly active in inhibiting the biofilm formation of the Gram-negative phytopathogen *P. carotovorum*. Indeed, at 1 μg/mL, it was capable of avoiding almost completely the biofilm formation (93.12%, Table 4) and to strongly affect the metabolic activity of the few biofilm cells (22.57% respect to control, Table 5). On the contrary, *L. petersoni* EO at a concentration of 2.0 μg/mL induced a relatively lower inhibition (66.67%, Table 4) on biofilm formation, while also markedly affecting metabolic activity (16.09% with respect to control) (Table 5). These results are in accordance with what was previously reported about antimicrobial properties of a hydroalcholic extract of *L. petersonii* leaves (Shirdashtzadeh et al., 2017).

### Phytotoxic Activity

A delay in germination or any other adverse effect on plants caused by specific substances is defined as phytotoxicity (Baumgarten and Spiegel, 2004). Moreover, secondary metabolites produced by plants, micro-organisms, viruses, and fungi, can alter the growth of the target species, with both positive and negative effects, through a phenomenon called “allelopathy” (Zeng et al., 2010).

Parameters to analyze the effects of natural or chemical substances on the growing of selected vegetal species are: relative or absolute germination and relative root elongation (Barral and Paradelo, 2011).

**FIGURE 6:**
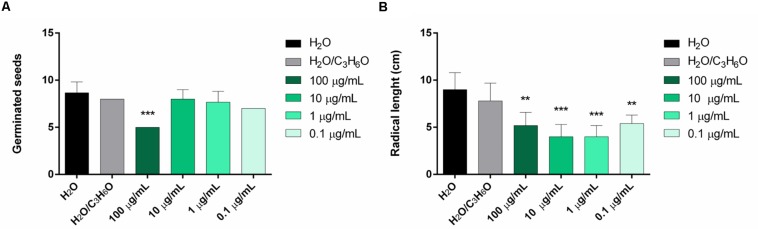
Phytotoxic activity of *L. petersonii* EO against germination of *R. sativus* **(A)** and radical elongation of *S. lycopersicum* **(B)**, 120 h after sowing. Results are the mean of three experiments ± SD. ** *p* < 0.01; *** *p* < 0.001 compared to control (ANOVA followed by Dunnett’s multiple comparison test).

In this study, the two EOs were evaluated for their activity against germination and radical elongation of radish, lettuce, garden cress, tomato, rye grass, and purslane. *L. petersonii* EO showed inhibitory activity against the germination of *R. sativus* (Figure 6A) and radical elongation of *S. lycopersicum* (Figure 6B). The treatment of seeds with a concentration of 100 μg/mL inhibited germination of *R. sativus*; all doses tested seemed to be active against radical elongation of *S. lycopersicum*. *E. gunnii* EO showed no phytotoxic activity on the tested seed (data not shown). Its principal component, 1,8 cineole, was inactive against several of the tested seeds, as shown in our previous studies (Caputo et al., 2018).

Few previous studies have reported data about the phytotoxicity and/or allelopathy of extracts and or EOs of plants belonging to the Myrtaceae family. Imatomi et al. (2013) studied the phytotoxic potential of leaf extracts of *Myrcia tomentosa* Glaz. and showed that ethyl acetate extract was the most active on the roots of lettuce, tomato, and onion, as well as on the shoots of lettuce and tomato. Instead, ethyl acetate extract of leaves of *Blepharocalyx salicifolius* (Kunth) had a high phytotoxic activity against *Triticum aestivum* L., *Sesamum indicum* L., *Echinochloa crusgalli* L. Beauv., and *Euphorbia heterophylla* L. (Habermann et al., 2015, 2016). Methanol extract of *Eugenia flavescens* DC leaves showed significant seed germination inhibition of *Mimosa pudica* and *Senna obtusifolia* (Cantanhede Filho et al., 2017). Moreover, *Callistemon viminalis* EO showed dose-dependent allelopathic activity on *L. sativa* seeds (de Oliveira et al., 2014). *Myrtus communis* L. EO inhibited seed germinations and seedling growths of *Amaranthus retroflexus* L., *Chenopodium album* L., *Cirsium arvense* (L.) SCOP., *Lactuca serriola* L., and *Rumex crispus* L. (Kordali et al., 2016).

In this study, we evaluated for the first time the phytotoxicity of *L. petersonii* EO; in fact, no previous studies have reported similar data about this or other plants from the genus *Leptospermum*.

On the contrary, the phytotoxic and allelopathic activities of several *Eucalyptus* species are well known in both natural and modified ecosystems (Chu et al., 2014) and some EOs from *Eucalyptus* spp. have been proposed for use in agricultural and forestry management (Ramezani et al., 2008). Despite the fact that the *Eucalyptus* genus is probably among the most investigated for its allelopathic properties, no studies are currently available on the activity of *E. gunnii* EO on seeds growing.

## Conclusion

This is the first study that provides a detailed SEM analysis of the leaf micromorphology of *L. petersonii* and *E. gunnii*. The quali-quantitative analyses showed a superimposable phytochemical profile for *E. gunnii* EO with respect to other previously published data. In addition, it highlighted the phytochemical composition of the EO of a quite rare *L. petersonii* chemical variety “B CT Australian Rose,” with a rose-like odor and containing geraniol and geranyl acetate as main constituents.

This is also the first time that the effects of these EOs on the biofilm formation biofilm cells metabolic activity were investigated.

This could open new perspectives for the application of *L. petersonii* and *E. gunnii* EOs.

Despite both EOs showing a good antimicrobial activity, the *E. gunnii* EO was the strongest one. This is probably due to the high content of 1,8-cineole, although a synergism with γ-terpinene and *trans*-sabinene hydrate-acetate could be postulated in light of previous literature data about the antimicrobial activity of the pure compound 1,8-cineole against the same pathogens.

In addition, both EOs were particularly effective against *S. aureus* biofilm formation, with *E. gunnii* EO showing the strongest activity not only with respect to *L. petersonii* EO, but also compared to other EOs isolated from other *Eucalyptus* species.

On the contrary, *L. petersonii* chemical variety “B CT Australian Rose” EO showed inhibitory activity against germination and radical elongation of *R. sativus* and *S. lycopersicum*, respectively, while no phytotoxic activity was detected for *E. gunnii* EO.

Considering these results, further investigations on these EOs could provide useful applications for both the treatment of human microbial infections and for agronomic management.

## Data Availability Statement

All datasets generated for this study are included in the article.

## Author Contributions

VD, FN, LaC, and DT designed the project. MV and GT distilled the EOs. FN and FF performed the antibacterial assays. LuC studied the phytotoxic activity and performed the data analysis. LaC carried out the micromorphological characterization. AS and LuC performed the chemical characterization. All authors contributed to writing and revising the manuscript.

## Conflict of Interest

GT is the owner of Essentially Australia. The remaining authors declare that the research was conducted in the absence of any commercial or financial relationships that could be construed as a potential conflict of interest.

## Abbreviations

DMSO: dimethylsulfoxide
EO(s): essential oil(s)
FE-SEM: field-emission—scanning electron microscopy
FID: flame ionization detector
GC: gas chromatography
MIC: minimal inhibitory concentration
MS: mass spectrometry
MTT: 3-(4,5-dimethyl-2-thiazolyl)-2,5-diphenyl-2H-tetrazolium bromide
NIST: National Institute of Standards and Technology
PBS: phosphate buffered saline
SD: standard deviation.

## References

[B1] Adams R. P. (2007). *Identification of Essential Oil Components by Gas Chromatography/Mass Spectroscopy.* Carol Stream, IL: Allured Publishing.

[B2] Barral M. T. Paradelo R. (2011). A review on the use of phytotoxicity as a compost quality indicator. *Dyn. Soil Dyn. Plant* 5 36–44.

[B3] Batish D. R. Singh H. P. Kohli R. K. Kaur S. (2008). Eucalyptus essential oil as a natural pesticide. *For. Ecol. Manag.* 256 2166–2174. 10.1016/j.foreco.2008.08.008

[B4] Baumgarten A. Spiegel H. (2004). *Phytotoxicity (Plant Tolerance).* Vienna: Agency for Health and Food Safety.

[B5] Bernal-Mercado A. T. Gutierrez Pacheco M. M. Encinas Basurto D. MataHaro V. Lopez Zavala A. A. Islas Osuna M. A. (2019). Synergistic mode of action of catechin, vanillic and protocatechuic acids to inhibit the adhesion of uropathogenic *Escherichia coli* on silicone surfaces. *J. Appl. Microbiol.* 128 387–400. 10.1111/jam.14472 31573730

[B6] Bewley J. D. (1997). Seed germination and dormancy. *Plant Cell* 9 1055–1066. 12237375 10.1105/tpc.9.7.1055 PMC156979

[B7] Brooker M. I. H. Kleinig D. A. (1996). *Eucalyptus*. *An Illustrated Guide to Identification.* Sidney, OH: Reed New Holland.

[B8] Brooker S. G. Cambie R. C. Cooper R. C. (1987). *New Zealand Medicinal Plants.* London: Heinemann.

[B9] Brophy J. J. Goldsack R. J. Punruckvong A. Bean A. R. Forster P. I. Lepschi B. J. (2000). Leaf essential oils of the genus *Leptospermum* (Myrtaceae) in eastern Australia, Part 7. *Leptospermum petersonii*, *L. liversidgei* and allies. *Flavour Fragr. J.* 15 342–351. 10.1002/1099-1026(200009/10)15:5<342::aid-ffj924>3.0.co;2-v

[B10] Bugarin D. Grbovixć S. Orèiè D. Mitić-Ćulafić D. Knežević-Vukèević J. Mimica-Dukić N. (2014). Essential oil of *Eucalyptus Gunnii* hook. As a novel source of antioxidant, antimutagenic and antibacterial agents. *Molecules* 19:19007. 10.3390/molecules191119007 25412046 PMC6271932

[B11] Cadena M. B. Preston G. M. Van der Hoorn R. A. L. Townley H. E. Thompson I. P. (2018). Species-specific antimicrobial activity of essential oils and enhancement by encapsulation in mesoporous silica nanoparticles. *Ind. Crops Prod.* 122 582–590. 10.1016/j.indcrop.2018.05.081

[B12] Camporese A. (2013). *In vitro* activity of *Eucalyptus smithii* and *Juniperus communis* essential oils against bacterial biofilms and efficacy perspectives of complementary inhalation therapy in chronic and recurrent upper respiratory tract infections. *Infez. Med.* 21 117–124. 23774975

[B13] Cantanhede Filho A. J. Santos L. S. Guilhon G. Zoghbi M. D. G. B. Ports P. S. Rodrigues I. (2017). Triterpenoids, phenolics and phytotoxic effects from *Eugenia flavescens* DC (Myrtaceae) leaves.Quim. *Nova* 40 252–259.

[B14] Caputo L. Nazzaro F. Souza L. Aliberti L. De Martino L. Fratianni F. (2017). *Laurus nobilis*: composition of essential oil and its biological activities. *Molecules* 22:930. 10.3390/molecules22060930 28587201 PMC6152719

[B15] Caputo L. Trotta M. Romaniello A. De Feo V. (2018). Chemical composition and phytotoxic activity of *Rosmarinus officinalis* essential oil. *Nat. Prod. Commun.* 13 1367–1370.

[B16] Carr D. Carr S. G. (1970). Oil glands and ducts in *Eucalyptus* L’Herit. II. Development and structure of oil glands in the embryo. *Aust. J. Bot.* 18 191–212.

[B17] Chieco C. Rotondi A. Morrone L. Rapparini F. Baraldi R. (2012). An ethanol-based fixation method for anatomical and micro-morphological characterization of leaves of various tree species. *Biotech. Histochem.* 88 109–119. 10.3109/10520295.2012.746472 23244233

[B18] Chu C. Mortimer P. E. Wang H. Wang Y. Liu X. Yu S. (2014). Allelopathic effects of Eucalyptus on native and introduced tree species. *For. Ecol. Manage.* 323 79–84. 10.1016/j.foreco.2014.03.004

[B19] Crowe A. (1997). *A Field Guide to the Native Edible Plants of New Zealand.* Auckland: Penguin Books.

[B20] Davies N. W. (1990). Gas chromatographic retention indices of monoterpenes and sesquiterpenes on methyl silicone and Carbowax 20M phases. *J. Chromatogr.* 503 1–24. 10.1016/s0021-9673(01)81487-4

[B21] de Oliveira C. M. das Graças Cardoso M. da Silva, Figueiredo A. C. de Carvalho M. L. M. de Miranda C. A. S. F. (2014). Chemical composition and allelopathic activity of the essential oil from *Callistemon viminalis* (myrtaceae) blossoms on lettuce (*Lactuca sativa* L.) seedlings. *Am. J. Plant Sci.* 5 3551–3557. 10.4236/ajps.2014.524371

[B22] Del Monte D. De Martino L. Marandino A. Fratianni F. Nazzaro F. De Feo V. (2015). Phenolic content, antimicrobial and antioxidant activities of *Hypericum perfoliatum* L. *Ind. Crop. Prod.* 74 342–347. 10.1080/13880209.2016.1270973 28147885 PMC6130493

[B23] Demuner A. J. Almeida Barbosa L. C. Gonçalves Magalhaes C. Da Silva C. J. Alvares Maltha C. R. Lelis Pinheiro A. (2011). Seasonal variation in the chemical composition and antimicrobial activity of volatile oils of three species of *Leptospermum* (Myrtaceae) grown in Brazil. *Molecules* 16 1181–1191. 10.3390/molecules16021181 21270734 PMC6259844

[B24] Dhakad A. K. Pandey V. V. Beg S. Rawat J. M. Singh A. (2018). Biological, medicinal and toxicological significance of Eucalyptus leaf essential oil:a review.J. *Sci. Food Agric.* 98 833–848. 10.1002/jsfa.8600 28758221

[B25] Djordjevic D. Wiedmann M. McLandsborough L. A. (2002). Microtiter plate assay for assessment of *Listeria monocytogenes* biofilm formation. *Appl. Environ. Microbiol.* 68 2950–2958. 10.1128/aem.68.6.2950-2958.2002 12039754 PMC123944

[B26] Dorman H. J. D. Deans S. G. (2000). Antimicrobial agents from plants: antibacterial activity of volatile plant oils. *J. Appl. Microbiol.* 88 308–316. 10.1046/j.1365-2672.2000.00969.x 10736000

[B27] Elaissi A. Marzouki H. Medini H. Larbi Khouja M. Farhat F. Lynene F. (2010). Variation in volatile leaf oils of 13 *Eucalyptus* species harvested from Souinet Arboreta (Tunisia). *Chem. Biodivers.* 7 909–921. 10.1002/cbdv.200900229 20397231

[B28] Elaissi A. Salah K. H. Mabrouk S. Larbi K. M. Chemli R. Harzallah-Skhiri F. (2011). Antibacterial activity and chemical composition of 20 Eucalyptus species’ essential oils. *Food Chem.* 129 1427–1434. 10.1016/j.foodchem.2011.05.100

[B29] Forrest M. (2002). The performance of a *Eucalyptus gunnii* cut foliage plantation over 7 years. *Irish J. Agr. Food Res.* 41 235–245.

[B30] Fratianni F. Cozzolino A. De Feo V. Coppola, R. Ombra M. N. Nazzaro F. (2019). Polyphenols, antioxidant, antibacterial, and biofilm inhibitory activities of peel and pulp of *Citrus medica* L., *Citrus bergamia*, and *Citrus medica* cv. Salò Cultivated in Southern Italy. *Molecules* 24:4577. 10.3390/molecules24244577 31847295 PMC6943604

[B31] Fratianni F. Ombra M. N. Cozzolino A. Riccardi R. Spigno P. Tremonte P. (2016). Phenolic constituents, antioxidant, antimicrobial and anti-proliferative activities of different endemic Italian varieties of garlic (*Allium sativum* L.). *J. Funct. Foods* 21 240–248. 10.1016/j.jff.2015.12.019

[B32] Gahlot R. Nigam C. Kumar V. Yadav G. Anupurba S. Gahlot R. (2014). Catheter-related bloodstream infections. *Int. J. Crit. Illn. Inj. Sci.* 4 162–167. 25024944 10.4103/2229-5151.134184 PMC4093967

[B33] Goodner K. L. (2008). Practical retention index models of OV-101, DB-1, DB-5, and DB-Wax for flavor and fragrance compounds. *LWT Food Sci. Technol.* 41 951–958. 10.1016/j.lwt.2007.07.007

[B34] Gutierrez-Pacheco M. M. Gonzalez-Aguilar G. A. Martinez-Tellez M. A. Lizardi-Mendoza J. Madera-Santana T. J. Bernal-Mercado A. T. (2018). Carvacrol inhibits biofilm formation and production of extracellular polymeric substances of *Pectobacterium carotovorum* subsp. carotovorum. *Food Control* 89 210–218. 10.1016/j.foodcont.2018.02.007

[B35] Habermann E. Imatomi M. De Cassia Pereira V. Cevithereza Pontes F. Gualtieri J. Cristina S. (2015). Phytotoxic activity of stem bark and leaves of *Blepharocalyx salicifolius* (Myrtaceae) on Weeds. *Acta Biolo. Colom.* 20 153–162. 10.15446/abc.v20n1.42756

[B36] Habermann E. Pontes F. C. Pereira V. C. Imatomi M. Gualtieri S. C. J. (2016). Phytotoxic potential of young leaves from *Blepharocalyx salicifolius* (Kunth) O. Berg (Myrtaceae). *Braz. J. Biol.* 76 531–538. 10.1590/1519-6984.24114 26959947

[B37] Hartmann R. Singh K. P. Pearce P. Mok R. Boya Song B. Francisco Díaz-Pascual F. (2019). Emergence of three-dimensional order and structure in growing biofilms. *Nat. Phys.* 15 251–256. 10.1038/s41567-018-0356-9 31156716 PMC6544526

[B38] Hendry E. R. Worthington T. Conway B. R. Lambert P. A. (2009). Antimicrobial efficacy of eucalyptus oil and 1,8-cineole alone and in combination with chlorhexidine digluconate against microorganisms grown in planktonic and biofilm cultures. *J. Antim. Chemoth.* 64 1219–1225. 10.1093/jac/dkp362 19837714

[B39] Henry-Stanley M. J. Hess D. J. Barnes A. M. T. Dunny G. M. Carol L. Wells C. L. (2010). Bacterial contamination of surgical suture resembles a biofilm. *Surg. Infect. (Larchmt)* 11 433–439. 10.1089/sur.2010.006 20673144 PMC2967823

[B40] Heras B. Scanlon M. J. Martin J. L. (2015). Targeting virulence not viability in the search for future antibacterials. *Br. J. Pharmacol.* 79 208–215. 10.1111/bcp.12356 24552512 PMC4309627

[B41] Hood J. R. Burton D. M. Wilkinson J. M. Cavanagh H. M. A. (2010). The effect of *Leptospermum petersonii* essential oil on *Candida albicans* and *Aspergillus fumigatus*. *Med. Mycol.* 48 922–931. 10.3109/13693781003774697 20446888

[B42] Hosseini Nezhad M. Alamshahi L. Panjehkeh N. (2012). Biocontrol efficiency of medicinal plants against *Pectobacterium carotovorum*, *Ralstonia solanacearum* and *Escherichia coli*. *Open Conf. Proc. J.* 3 46–51. 10.2174/1876326x01203020046

[B43] Huth M. A. Huth A. Koch K. (2018). Morphological diversity of β-diketone wax tubules on *Eucalyptus gunnii* leaves and real time observation of self-healing of defects in the wax layer. *Aust. J. Bot.* 66 313–324.

[B44] Imatomi M. Novaes P. Matos A. P. Gualtieri S. C. Molinillo J. M. Lacret R. (2013). Phytotoxic effect of bioactive compounds isolated from *Myrcia tomentosa* (Myrtaceae) leaves. *Biochem. Syst. Ecol.* 46 29–35. 10.1016/j.bse.2012.09.005

[B45] Jennings W. Shibamoto T. (1980). *Qualitative Analysis of Flavour and Fragrance Volatiles by Glass Capillary Gas Chromatography.* New York, NY: Academic Press.

[B46] Johnson C. T. (1980). The leaf anatomy of *Leptospermum* Forst. (Myrtaceae). *Aust. J. Bot.* 28 77–104.

[B47] Junka A. Żywicka A. Chodaczek G. Dziadas M. Czajkowska J. Duda-Madej A. (2019). Potential of biocellulose carrier impregnated with essential oils to fight against bioflms formed on hydroxyapatite. *Sci. Rep* 9:1256. 10.1038/s41598-018-37628-x 30718663 PMC6362291

[B48] Kairo S. K. Bedwell J. Tyler P. C. Carter A. Corbel M. J. (1999). Development of a tetrazolium salt assay for rapid determination of viability of BCG vaccines. *Vaccine* 17 2423–2428. 10.1016/s0264-410x(99)00023-7 10392624

[B49] Karpanen T. J. Worthington T. Hendry E. R. Conway B. R. Lambert P. A. (2008). Antimicrobial efficacy of chlorhexidine digluconate alone and in combination with eucalyptus oil, tea tree oil and thymol against planktonic and biofilm cultures of *Staphylococcus epidermidis*. *J Antimicrob. Chemother.* 62 1031–1036. 10.1093/jac/dkn325 18703525

[B50] Kim E. Park I. K. (2012). Fumigant antifungal activity of Myrtaceae essential oils and constituents from *Leptospermum petersonii* against three *Aspergillus* species. *Molecules.* 17 10459–10469. 10.3390/molecules170910459 22945026 PMC6268886

[B51] Kordali S. Usanmaz A. Cakir A. Komaki A. Ercisli S. (2016). Antifungal and herbicidal effects of fruit essential oils of four *Myrtus communis* genotypes. *Chem. Biodivers.* 13 77–84. 10.1002/cbdv.201500018 26765354

[B52] Kubheka G. C. Coutinho T. A. Moleleki N. Moleleki L. N. (2013). Colonization patterns of an mCherry-tagged *Pectobacterium carotovorum* subsp. brasiliense strain in potato plants. *Phytopathology* 103 1268–1279. 10.1094/PHYTO-02-13-0049-R 23758294

[B53] Lee B. H. Annis P. C. Tumaalii F. Choi W. S. (2004). Fumigant toxicity of essential oils from the Myrtaceae family and 1,8-cineole against 3 major stored-grain insects. *J. Stored Prod. Res.* 40 553–564. 10.1016/j.jspr.2003.09.001

[B54] Lis-Balchin M. Deans S. Hart S. (1996). Bioactivity of New Zealand medicinal plant essential oils. *Acta Hortic.* 426 13–30. 10.17660/actahortic.1996.426.1

[B55] Lis-Balchin M. Deans S. G. (1997). Bioactivity of selected plant essential oils against *Listeria monocytogenes*. *J. Appl. Bacteriol.* 82 759–762. 10.1046/j.1365-2672.1997.00153.x 9202441

[B56] Lis-Balchin M. Hart S. L. Deans S. G. (2000). Pharmacological and antimicrobial studies on different tea-tree oils (*Melaleuca alternifolia*, *Leptospermum scoparium* or manuka and *Kunzeaericoides*or kanuka), originating in Australia and New Zealand. *Phytother. Res.* 14 623–629. 10.1002/1099-1573(200012)14:8<623::aid-ptr763>3.0.co;2-z 11114000

[B57] Lucia A. Licastro S. Zerba E. Masuh H. (2008). Yield, chemical composition, and bioactivity of essential oils from 12 species of *Eucalyptus* on *Aedes aegypti* larvae. *Entomol. Exp. Appl.* 129 107–114. 10.1111/j.1570-7458.2008.00757.x

[B58] Mabberley D. J. (1997). *The Plant-Book: A Portable Dictionary of the Vascular Plants.* Cambridge: Cambridge University Press.

[B59] MacRae J. M. Dipchand C. Matthew Oliver M. Moist L. Yilmaz S. Lok C. (2016). Arteriovenous access infection, neuropathy, and other complications. *Can. J. Kidney Health Dis.* 3 1–13. 10.1177/2054358116669127 28270919 PMC5332082

[B60] Mehrsorosh H. Gavanji S. Larki B. Mohammadi M. D. Karbasiun A. Bakhtari A. (2014). Essential oil composition and antimicrobial screening of some Iranian herbal plants on *Pectobacterium carotovorum*. *Glob. NEST J.* 16 240–251. 10.30955/gnj.001205

[B61] Merghni A. Noumi E. Hadded O. Dridi N. Panwar H. Ceylan O. (2018). Assessment of the antibiofilm and antiquorum sensing activities of *Eucalyptus globulus* essential oil and its main component 1,8-cineole against methicillin-resistant *Staphylococcus aureus* strains. *Microb. Pathog.* 118 74–80. 10.1016/j.micpath.2018.03.006 29522803

[B62] Migacz I. P. Raeski P. A. Paes de Almeida V. Raman V. Nisgoski S. Bolzón de Muniz G. I. (2018). Comparative leaf morpho-anatomy of six species of Eucalyptus cultivated in Brazil. *Rev. Bras. Farmacogn.* 28 273–281. 10.1016/j.bjp.2018.04.006

[B63] Murugan K. Selvanayaki K. Al-Sohaibani S. (2016). Urinary catheter indwelling clinical pathogen biofilm formation, exopolysaccharide characterization and their growth influencing parameters Saudi. *J. Biol. Sci.* 23 150–159. 10.1016/j.sjbs.2015.04.016 26858552 PMC4705282

[B64] Nazzaro F. Fratianni F. De Martino L. Coppola R. De Feo V. (2013). Effect of essential oils on pathogenic bacteria. *Pharmaceuticals* 6 1451–1474. 10.3390/ph6121451 24287491 PMC3873673

[B65] Nuadha T. (2011). *Leptospermum Petersonii.* Lect Publishing.

[B66] O’Toole G. A. Kolter R. (1998). Flagellar and twitching motility are necessary for *Pseudomonas aeruginosa* biofilm development. *Mol. Microbiol.* 30 295–304. 10.1046/j.1365-2958.1998.01062.x 9791175

[B67] Park H. M. Kim J. Chang K. S. Kim B. S. Yang Y. J. Kim G. H. (2011). Larvicidal activity of Myrtaceae essential oils and their components against *Aedes aetypti*, acutetoxicity on *Daphnia magna*, and aqueous residue. *J. Med. Entomol.* 48 405–410. 10.1603/me10108 21485381

[B68] Purwatiningsih, Heather N. E. Hassan E. (2012). Efficacy of *Leptospermum petersonii* oil, on *Plutella xylostella*, and its parasitoid, *Trichogramma pretiosum*. *J. Econ. Entom.* 105 1379–1384. 10.1603/ec11382 22928319

[B69] Quatrin P. M. Verdi C. M. de Souza M. E. de Godoi S. N. Klein B. Gundel A. (2017). Antimicrobial and antibiofilm activities of nanoemulsions containing *Eucalyptus globulus* oil against *Pseudomonas aeruginosa* and *Candida* spp. *Microb. Pathog.* 112 230–242. 10.1016/j.micpath.2017.09.062 28970174

[B70] Ramezani S. Saharkhiz M. J. Ramezani F. Fotokian M. H. (2008). Use of essential oils as bioherbicides. *J. Essent. Oil Bear.Plants* 1 319–327. 10.1080/0972060X.2008.10643636

[B71] Riley M. (1994). *Mâori Healing and Herbal: New Zealand Ethnobotanical Sourcebook.* Paraparaumu: Viking Seven seas.

[B72] Rolim de Almeida L. F. Fernando Frei F. Mancini E. De Martino L. De Feo V. (2010). Phytotoxic activities of mediterranean essential oils. *Molecules* 15 4309–4323. 10.3390/molecules15064309 20657443 PMC6257658

[B73] Salehi B. Sharifi-Rad J. Quispe C. Llaique H. Villalobos M. Smeriglio A. (2019). Insights into *Eucalyptus* genus chemical constituents, biological activities and health-promoting effects. *Trends Food Sci. Technol.* 91 609–624. 10.1016/j.tifs.2019.08.003

[B74] Salem M. Z. M. Ashmawy A. N. Elansary H. O. El-Settawy A. A. (2015). Chemotyping of diverse *Eucalyptus* species grown in Egypt and antioxidant and antibacterial activities of its respective essential oils. *Nat. Prod. Res.* 29 681–685. 10.1080/14786419.2014.981539 25421867

[B75] Sambyal S. S. Sharma P. Shrivastava D. (2017). Anti-biofilm activity of selected plant essential oils against *Pseudomonas aeruginosa* and *Staphylococcus aureus*. *Int. J. Curr. Microbiol. Appl. Sci.* 6 444–450. 10.20546/ijcmas.2017.603.051

[B76] Sarker S. D. Nahar L. Kumarasamy Y. (2007). Microtitre plate-based antibacterial assay incorporating resazurin as an indicator of cell growth, and its application in the in vitro antibacterial screening of phytochemicals. *Methods* 42 321–324. 10.1016/j.ymeth.2007.01.006 17560319 PMC1895922

[B77] Sebei K. Sakouhi F. Herchi W. Larbi Khouja M. Boukhchina S. (2015). Chemical composition and antibacterial activities of seven *Eucalyptus* species essential oils leaves. *Biol. Res.* 48:7. 10.1186/0717-6287-48-7 25654423 PMC4417289

[B78] Shepherd T. Griffiths D. W. (2006). The effects of stress on plant cuticular waxes. *New Phytol.* 171 469–499. 10.1111/j.1469-8137.2006.01826.x 16866954

[B79] Shirdashtzadeh M. Chandrasena G. I. Henry R. McCarthy D. T. (2017). Plants that can kill; improving *E. coli* removal in stormwater treatment systems using Australian plants with antibacterial activity. *Ecol. Eng.* 107 120–125. 10.1016/j.ecoleng.2017.07.009

[B80] Van Vuuren S. F. Docrat Y. Kamatou G. P. P. Viljoen A. M. (2014). Essential oil composition and antimicrobial interactions of understudied tea tree species. *S. Afr. J. Bot.* 92 7–14. 10.1016/j.sajb.2014.01.005

[B81] Wiley (1998). *The Wiley Registry of Mass Spectral Data, with NIST Spectral Data CD Rom.* New York, NY: John Wiley & Sons.

[B82] Windsor S. A. M. Brooks P. (2012). Essential oils from *Leptospermums* of the sunshine coast and northern rivers regions. *Chem. Cent. J.* 6:38. 10.1186/1752-153X-6-38 22559035 PMC3418194

[B83] Zeng R. S. Mallik A. U. Luo S. (2010). *Allelopathy in Sustainable Agriculture and Forestry.* New York, NY: Springer Verlag.

